# Impact of land cover and landfills on the breeding effect and nest occupancy of the white stork in Poland

**DOI:** 10.1038/s41598-021-86529-z

**Published:** 2021-03-31

**Authors:** Joanna T. Bialas, Łukasz Dylewski, Andrzej Dylik, Tomasz Janiszewski, Ireneusz Kaługa, Tomek Królak, Robert Kruszyk, Krzysztof Pawlukojć, Zuzanna Pestka, Michał Polakowski, Adam Zbyryt, Marcin Tobolka

**Affiliations:** 1 Department of Zoology, Poznań University of Life Sciences, Wojska Polskiego 71C, 60-625 Poznań, Poland; 2 Institute of Dendrology, Polish Academy of Sciences, Parkowa 5, 62-035 Kórnik, Poland; 3 Kotwicowa 15, 85-435 Bydgoszcz, Poland; 4 Department of Biodiversity Studies and Bioeducation, Faculty of Biology and Environmental Protection, University of Łódź, Banacha 1/3, 90-237 Łódź, Poland; 5 Grupa EkoLogiczna, B. Chrobrego 15/83, 08-110 Siedlce, Poland; 6 Towarzystwo Przyrodnicze “Alauda”, M. Skłodowskiej-Curie 65, 87-100 Toruń, Poland; 7 Piotrowicka 52A, 44-341 Skrbeńsko, Poland; 8 Królowej Jadwigi, 18C/5, 11-500 Giżycko, Poland; 9 Vertebrate Ecology and Zoology Unit, University of Gdańsk, Bażyńskiego 8, 80-309 Gdańsk, Poland; 10 Department of Ecology and Anthropology, Institute of Biology, University of Szczecin, Wąska 13, 71-412, Szczecin, Poland; 11 The Polish Society for Bird Protection (PTOP), Ciepła 17, 15-471 Białystok, Poland; 12 Faculty of Biology, University of Białystok, Ciołkowskiego 1J, 15-245 Białystok, Poland; 13 Konrad Lorenz Institute of Ethology, Veterinary Medicine University of Vienna, Savoyenstraße 1, 1160 Wien, Austria

**Keywords:** Behavioural ecology, Population dynamics, Behavioural ecology, Population dynamics, Ecology, Zoology, Ecology

## Abstract

Food wastes are among the factors with the greatest effects on animal populations. The white stork is among bird species that clearly profit from feeding at landfills, at least in Western Europe and North Africa. However, the rate and the consequences of this feeding are still unknown in the Central-Eastern European population, which differs from the western population not only in terms of migration routes but also in the greater availability of suitable natural breeding habitats due to less intensified agriculture. The aim of the study was to describe the use of landfills and its consequences in terms of probability of nest occupation and breeding effects in different regions of Poland. Although the most important factors influencing nest-site selection and breeding effect are still habitat quality and weather conditions, distance to landfills is important in selection of nest sites. White storks use landfills most intensively late in the breeding season, independently of the density of breeding pairs. The results suggest that the use of landfills is not currently essential in the Central-Eastern European population of the white stork, does not affect breeding effect, and may be more frequent in non-breeders. However, this phenomenon is still developing and requires continuous monitoring.

## Introduction

The tremendous human impact on the environment has caused the decline of many animals due to severe changes, such as deforestation^1^, agricultural intensification^2^, and urbanisation^3^. Whereas many species are declining or even becoming extinct, some are capable of adapting and expanding their populations^4^, mainly due to evolutionary rescue^5^ or phenotypic plasticity^6^. In parallel to anthropogenic changes in land surface, human-produced food wastes provided to animals are among the most important factors influencing population dynamics, food webs, and inter- and intraspecies interactions of wild species living in anthropogenic habitats^7^. The problem of food waste production continues to grow and to affect biodiversity^7^.

The most intensely studied group of animals in terms of landfill use is composed of birds^8^. Birds foraging on landfills have been widely studied in many parts of the world^9,10^. Foraging at landfills has many advantages for birds, including spatiotemporal predictability of food sources^7^ and the high energetic value of the relevant food^11^, which may enhance breeding success^9,12,13^. Food wastes available at landfills, however, pose threats to foraging birds, such as the ingestion of solid waste, e.g. fragments of plastic, rubber, glass, or metal^14^, pathogens^15,16^, and toxins (e.g. heavy metals)^15,17,18^. Thus, ingestion of wastes may lead to choking, injury, or poisoning^14,15^. To date, 54 bird species have been recorded and reported as foraging on landfills around the globe^8^. Many of these species have increased in terms of abundance^8^. The most common species are from the following families: Corvidae, Laridae, Accipitridae, Ciconiidae, and Cathartidae.

The white stork *Ciconia ciconia* is one of the bird species that has benefitted from anthropogenic changes in the environment^19^; however, due to rapid large-scale environmental modification, this species has lacked suitable natural habitats. Changes in land management in Europe have progressed at different rates, i.e. Western Europe has been characterised by more rapid development of large-scale agriculture, while Eastern Europe has maintained less intensified agricultural production with a large proportion of natural and semi-natural habitats, as reflected in trends of farmland birds in Europe^20^. Moreover, white storks in Western and Central-Eastern Europe (CEE) belong to different migratory populations characterised by different migration routes^21^. Agricultural intensification on the breeding grounds and severe droughts on the wintering grounds were the most probable causes of the drop in the population of the white stork in Western Europe, which nearly led to the extinction of the species in some regions^19,22,23^. In recent years, the Western European population of the white stork has recovered, as a result of changes in foraging behaviour^24^. As an opportunistic species, white storks use the most abundant and most easily obtained food^25^. Use of anthropogenic food sources available from landfills and slaughterhouses, which caused shortening or aborting migration to wintering grounds, contributed to population growth^13,24^. The phenomenon of white storks foraging at landfills has been extensively described in Western Europe and North Africa^9,13,17,26,27^. In CEE, however, whereas such behaviour has already begun, it has not been studied comprehensively, except for the non-breeding season^28^. The use of landfills during breeding is still relatively rare, and thus has not been extensively studied^29^. However, a recent study of the CEE population indicates a visible trend towards nesting closer to landfills^30^, which may suggest that the use of landfills as a food source, and lead to increases in white stork populations in the future.

The actual scale of this phenomenon is still unknown. This knowledge may alter the existing view of white storks as indicators of the biodiversity and naturalness of the agricultural landscape^31^. White storks, as one of many opportunistic species of birds^32–35^, are capable of using landfills as feeding areas. It is worth assessing the scale of the phenomenon and determining whether it is connected with population density, habitat quality, or reproductive success. What is more, according to European Union regulations (Landfill Waste Council European Directive 1999/31/CE and Directive 2018/850/EC) open landfills will have been closed or covered soon. To estimate the effect of landfills closing for entire European white stork population more evidence is needed.

In this study we aimed to assess the scale of foraging by white storks at landfills and the resulting influence on the breeding ecology of eastern migratory white storks. Assuming that landfills have become important foraging grounds, we hypothesise that (i) white storks choose nests located close to landfills to better access to the plentiful foraging areas; (ii) white storks breeding closer to landfills experience a greater breeding effect (number of fledglings); and (iii) along with distance to a landfill, both nest occupation probability and breeding effect are affected by habitat quality and weather conditions. We also expect that (iv) the number of adult white storks foraging at landfills is correlated with the density of the white stork breeding population, and that (v) the number of white storks, as well as their age structure changes within the annual life cycle (breeding, post-breeding, autumn migration).

## Methods

### Study area

**Figure 1:**
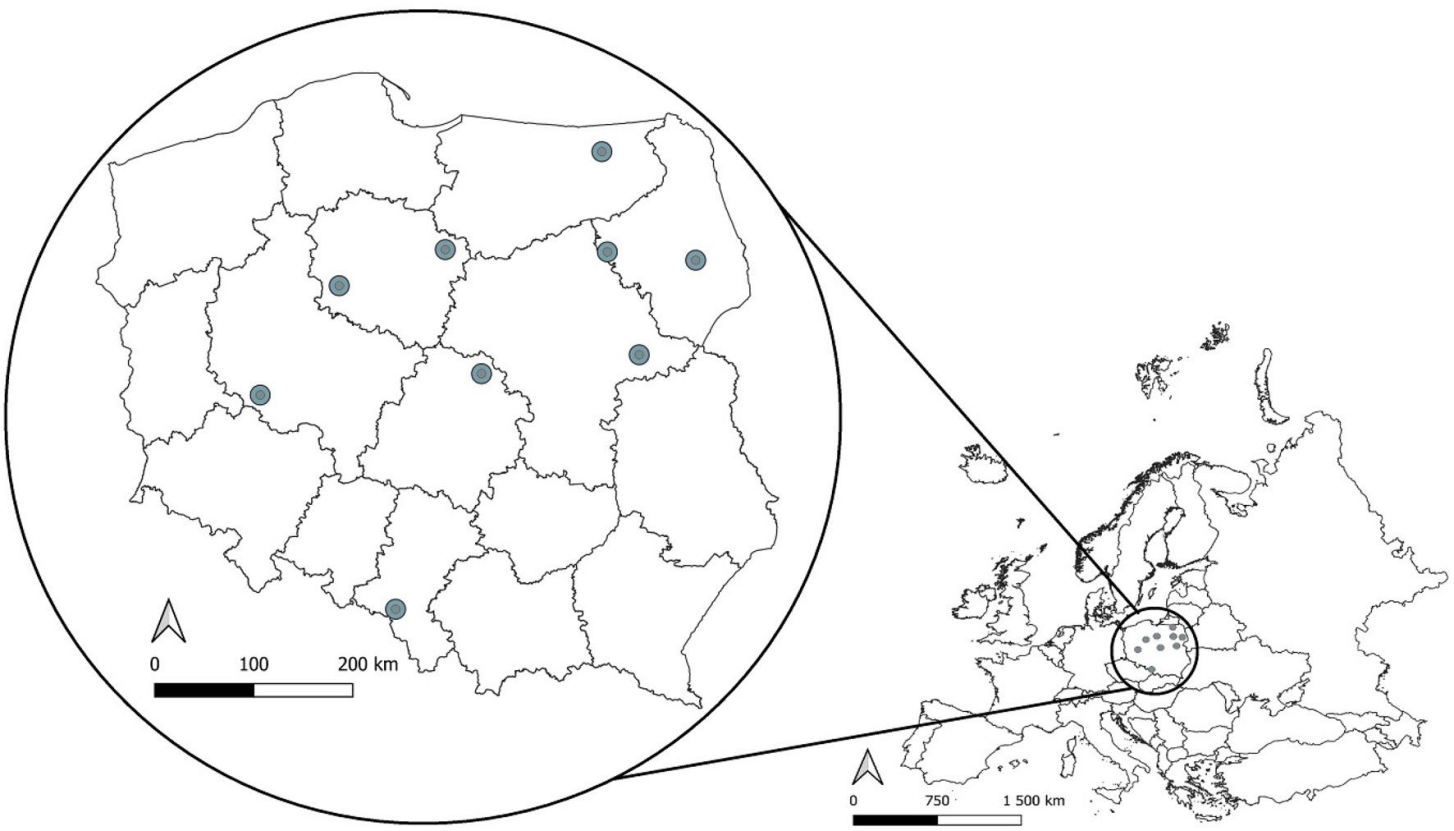
Map of Europe, with the location of studied landfills and censused areas around them (green dots). Data obtained from Eurostat (https://ec.europa.eu/eurostat/web/gisco/geodata/reference-data/administrative-units-statistical-units/countries) and Head Office of Geodesy and Cartography (http://www.gugik.gov.pl/pzgik/dane-bez-oplat/dane-z-panstwowego-rejestru-granic-i-powierzchni-jednostek-podzialow-terytorialnych-kraju-prg) under condition of non-commercial use. Figure generated with QGIS 3.18.0 (https://qgis.org/en/site/index.html).

The study was conducted at 9 landfills in Poland, Brodnica (53.233366 N, 19.374786 E), Czartoria (53.179539 N, 21.826949 E), Hryniewicze (53.065083 N, 23.153928 E), Jastrzębie-Zdrój (49.967990 N, 18.647621 E), Łowicz-Jastrzębia (52.103711 N, 19.900442 E), Spytkowo (54.090423 N, 21.803944 E), Trzebania (51.890007 N, 16.642788 E), Wawrzynki (52.899419 N, 17.776417 E), and Wola Suchożebrska (52.234621 N, 22.238896 E) (Fig. 1), as well as within the 10-km buffers around the central points of these landfills. The minimal distance between landfills was 90 km, which is significantly greater than the home range of breeding white storks^36^. Western and Eastern Poland differ in terms of both white stork population density and land use. Eastern Poland is rich in extensively cultivated lands, with many meadows and pastures^37^, whereas farming in Western Poland is rather intensive and rich in arable fields^38^. It has been clearly shown that the type of land cover determines the diet of white stork populations^39^. Regional differences are also expressed in the population trends of other bird species linked to agricultural environments^20^.

### Data collection

We visited landfills to record the presence of white stork individuals. We collected data in three phenological periods: breeding season, i.e. April, May, June, and the first half of July; post-breeding period, i.e. the second half of July and beginning of August; and autumn migration, i.e. after 5 August. We visited each landfill between 5 and 31 times during the season, at 2-week intervals, from April to August 2016. We conducted observations either from a single point, from which an observer was able to see the entire landfill, or from several points, in order to cover the entire area and to avoid missing any individuals. We started observations at least 2 h after sunrise and continued them for a minimum of 5 h (or even during the entire day) to cover the late morning, midday, and afternoon periods. We always recorded the starting and finishing time and the presence of white stork individuals. We noted the age (1: juvenile; 2: after the first calendar year) of each white stork individual and its time of arrival at and departure from the landfill if possible. We calculated the number of birds visiting a given landfill per hour of observations. As our data were collected through five months, and day elongated significantly, we chose to operate not on time of the day but on hours after sunrise.

In addition to observations at landfills, we monitored white stork nests within a radius of 10 km around each landfill and recorded the coordinates of each nest. We chose 10 km radius to avoid moving towards another landfill and to enable using consistent methodology of data collection, due to differences in density of white storks’ pairs among regions. In the first half of July, just before fledging, we recorded the number of fledglings standing on the nests and considered able to fly, according to a standard census method used with white storks^19^. We defined the number of fledglings as a measure of breeding effect in this study. In cases of nests without breeding success, we conducted additional interviews about the cause of breeding failure and pair history with farmers and hosts, in order to clarify the status of the nest. This is a reliable method thanks to the high level of social interest in the white stork^40^. From the census data we obtained information concerning the densities of breeding pairs per square km. We also used census data collected in previous and subsequent years (2009–18) from four areas (around the Łowicz-Jastrzębia, Trzebania, Wola Suchożebrska, and Brodnica landfills) and data from the area around the Wawrzynki landfill in 2016 and 2017, all collected according to the same standard methodology. We recorded occupation status of 1836 nests in total (118–537 nests per year), and the breeding effect of 1607 successful nests in total (100–448 nests per year). Moreover, we included data on weather conditions in each month of each year of the study (2009–18): average monthly minimal temperature, accumulated monthly precipitation, soil moisture at the end of each month, and monthly climatic water deficit (difference between monthly reference evapotranspiration and actual evapotranspiration) in the months March–July, obtained from TerraClimate, http://www.climatologylab.org/terraclimate.html^41^.

### Spatial analyses

For all spatial analyses, we used QGIS 2.18.13 open-source software. We calculated the distance from each nest to the centre of the nearest landfill and obtained information on the land cover within the home range (a radius of 2 km, based on Zurell et al.^36^, Nowakowski^43^) of each nest, using CORINE Land Cover 2012. Because white storks show a preference for a particular type of habitat, i.e. meadows, pastures, and wetlands, and avoid great forest complexes^31,43,44^, we used land cover data as a proxy for habitat quality in this study (details in Bialas et al.^30^). This choice is supported by the study of the relationship between diet and land cover, which has clearly shown that such data can be used to investigate the links between various landscape traits and breeding effects in breeding white storks^39^.

We obtained land cover data from the website of the Chief Inspectorate of Environmental Protection (http://www.eea.europa.eu/data-and-maps). We used the processing plug-in for QGIS to analyse the share of 20 land cover classes in buffers characterised by radii of 2 km created around each nest, which is described as the most intensively used are of home range^36,42^. We categorised CORINE Land Cover classes into seven groups appropriate for this study: areas greatly altered by humans (including continuous urban fabric, discontinuous urban fabric, industrial or commercial units, mineral extraction sites, construction sites, green urban areas, sport and leisure facilities); non-irrigated arable land; other agricultural land (fruit trees and berry plantations, complex cultivation patterns, and land principally occupied by agriculture, with significant areas of natural vegetation); pastures and meadows; forests (broad-leaved forests, coniferous forests, mixed forests, transitional woodland-shrub); inland marshes; and inland waters (water courses, water bodies). We calculated percentage of each land cover type in the 2-km buffer around each nest.

### Data processing and statistical analyses

We analysed the impacts of potential predictors on nest occupation using machine learning techniques for data collections covering one year (2016) from nine sites, 2 years from one site, and 10 years from four sites. As the amount of data for 2016 was significantly greater, and controls on the landfills were conducted in 2016 we chose to analyse it separately. To avoid multicollinearity, we excluded three variables (shares of forests, inland marshes, and inland waters) from both models. Multicollinearity in the remaining explanatory variables was not excessive (VIF < 2). In the structures of both models, we included nine continuous predictors: average minimum temperature during breeding season, i.e. March-July (temp), average climatic water deficit during breeding season (def), average soil moisture at the end of each month during breeding season (soil), average accumulated monthly precipitation during breeding season (ppt), distance to landfill (distance, which was natural log-transformed), share of areas greatly altered by humans (human), non-irrigated arable land (arable), other agricultural land (agri), and pastures and meadows (grassland). In the model for the 10-year data survey, we included two additional category predictors: year and site. This method was chosen due to the poor representation and low level of variation of nest occupation, i.e. nests occupied or not occupied within the site for each year (occupation rate 0.74–1 per study site); methods based on generalised linear mixed-effects models would not have been sensitive enough^45^. Machine learning approaches, supported by explanatory tools^46^, enable the objective generation of rules of prediction, with insight into variable importance and partial dependences, assuming mean values for all other predictors. In our model we used nest occupation as a binary response variable (0: non-occupation; 1: occupation). Prior to model development, we divided dataset into training (75% of observations) and test (25%) sets, with an equal proportion of positive and negative observations. Test sets were not used in model building. We implemented (during model development) internal repeated cross-validation (10 repeats, 10 times) to reduce model overfitting^47^. We choose three methods—random forests (RF), support vector machines (SVM), and gradient boosted modelling (GBM)—as the best analytic approach to testing. The model selection was based on AUC (area under receiver-operator curve) values. AUC takes values from 0 to 1, whereas a value of 0.5 indicates random selection (minimum model performance). As a final model, we chose GBM, with the highest AUC assessed using an independent test set (25% of observations) in order to prevent model overfitting. We assessed differences among models using two R packages for the machine learning model: DALEX^46^ and ceterisParibus^46^. We calculated the importance of variables using loss-drop of RMSE (root mean squared error), i.e. an increase in predicted RMSE when a particular predictor is perturbed within a dataset. The higher the increment of RMSE, the more important the variable in proper prediction of the outcome. As a reference value we used loss-drop of the full model, i.e. the increment of RMSE when all predictors are randomly perturbed^46^. We evaluated the impact of particular predictors on modelled output using Ceteris Paribus plots, i.e. predicted nest occupation for each predictor assuming constant (mean) value of all remaining predictors. We conducted independent analysis for the year 2016 alone, for which we had obtained the most comprehensive dataset (all nine study plots). We decided to analyse it separately as well, to study if the chosen factors can influence the nest occupation in a different manner than when we take all years together.

To determine which predictors influenced the white stork breeding effect, we used a linear mixed effect model implementing a restricted maximum-likelihood (REML) estimator. As a response variable, we used number of fledglings, along with the following explanatory variables: average minimum temperature during breeding season, i.e. March-July (temp), average climatic water deficit during breeding season (def), average soil moisture at the end of each month during breeding season (soil), average accumulated monthly precipitation during breeding season (ppt), distance to landfill (dist_land, which was natural log-transformed), share of areas greatly altered by humans (human), non-irrigated arable land (arable), other agricultural land (agri_land), and pastures and meadows (meadow). We also included quadratic terms for non-irrigated arable and other agricultural land to allow for a nonlinear relationship in both models, as supported by improvement of the model AICc score (Supplementary Table S1). We used two random effects: year and nest ID nested within site.

To determine which factors influence the frequency of white stork visits to landfills, we used a generalised additive mixed model (GAMM) with negative binomial distribution. The model included numbers of white storks observed per hour as a dependent variable and landfill ID as a random factor to control if the foraging on landfill is not characteristic for local populations^16^. As explanatory variables, we used population density and landfill area [ha], because the landfill foraging frequency may be dependent on distance between breeding pair, which theoretically may affect the copying behaviour by individuals. We also included the following smooth-term explanatory variables with the third-degree polynomial: time after sunrise, day of year, and temperature to test our hypotheses. Our observations suggested that the time of day is non-random for white storks’ use of landfills. Time of the season may also affect the numbers of storks using the landfills, as later in the breeding season not only nestlings' nutrition demands grow but also the number of non-breeders has its peak. We used third-degree polynomial because, based on already published work^16^, white stork populations and individuals may differ in extent of using landfills as foraging grounds, and this behaviour may be locally specific. The third-degree polynomial also better fits the curves.

For all statistical analyses we used R 4.0.0 (R Core Developmental Team, 2020). We carried out the machine learning technique using the caret^47^, randomForest^48^, gbm^49^, and e1071^50^ packages. We used the lme4 package for the GLMM^51^, and the MuMIn package for model selection^52^. We carried out the GAMM using the gamm4 package^53^. For the graphical visualisation we used the ggplot2 package^54^.

## Results

Population densities varied between 0.02 and 0.29 pairs per square kilometre, with average of 0.1 breeding pairs per square kilometre. Occupation rate ranged from 0.84 to 0.94 per year, from 0.74 to 1 per site. Breeding effects varied through years from a minimum number of 0 to a maximum number of 5, with average number of 2.07 fledglings per breeding pair.

**Figure 2:**
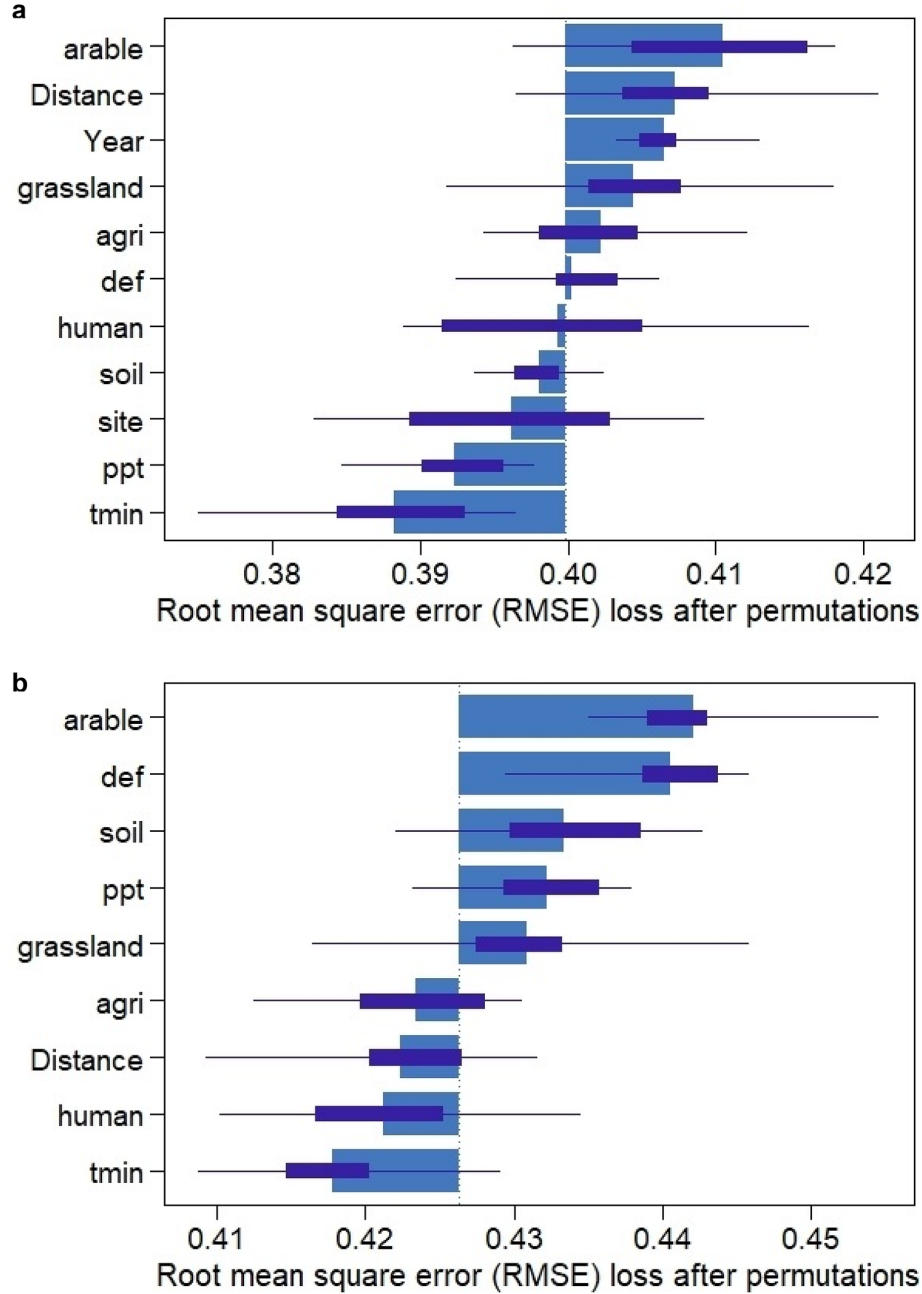
Importance of variables in models of nest occupation in years 2009–2018 **(a)** and 2016 **(b)**. Importance is expressed by loss-drop in RMSE. Dotted lines refer to loss-drop after perturbation of all predictors, i.e. the larger the bar, the higher the importance of a particular variable. Importance below dotted lines indicates variables with lower importance than random perturbation of all variables within a dataset. Abbreviations used in the table: arable—cover of non-irrigated arable lands, distance—ln distance to the nearest landfill, grassland—cover of pastures and meadows, agri—cover of other agricultural lands, def—average water deficit during breeding season, human—cover of highly human changed areas, soil moisture—average soil moisture at the end of each month during breeding season, site—site ID, ppt—average accumulated monthly precipitation during breeding season, tmin—average minimum temperature during breeding season.

**Figure 3:**
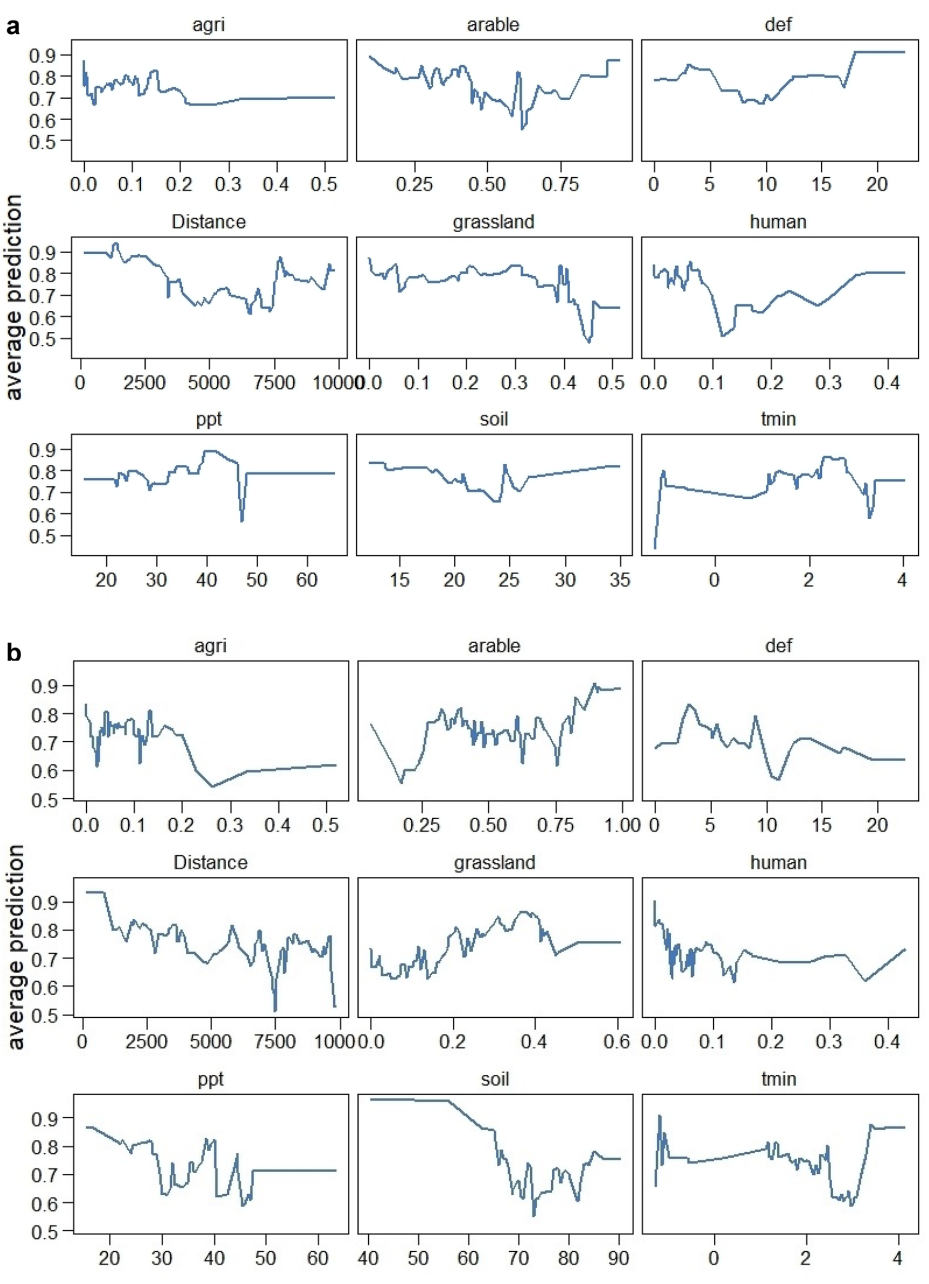
Partial dependence plots showing average predictions of nest occupation in years 2009–2018 **(a)** and for one season (2016) **(b)**. Lines show average predictions for continuous predictors, assuming mean values of all remaining predictors. For categorical predictors we showed mean predicted survival probability assuming constant level of other predictors for each group. Abbreviations used in the table: agri—cover of other agricultural lands, arable—cover of non-irrigated arable lands, def—average water deficit during breeding season, distance—ln distance to the nearest landfill, grassland—cover of pastures and meadows, human—cover of highly human changed areas, ppt—average accumulated monthly precipitation during breeding season, soil moisture—average soil moisture at the end of each month during breeding season, tmin—average minimum temperature during breeding season.

The model including all data for the period 2009–18 revealed that the most important variables for nest occupation probability were: cover of non-irrigated arable lands, distance to landfill, year, cover of pastures and meadows, and other agricultural lands (Figs. 2a, 3a). The average prediction indicated that the most important variables, i.e. non-irrigated arable lands and distance to landfill, were negatively correlated with the probability of nest occupation. The model for occupation probability in 2016 showed that the most important variables were cover of non-irrigated arable lands, climatic water deficit, soil moisture, precipitation, and cover of pastures and meadows (Figs. 2b, 3b). The average prediction indicated that the most important variables, i.e. climatic water deficit and soil moisture, were negatively correlated and that non-irrigated arable lands exerted a slight negative effect on probability of nest occupation.

**Figure 4:**
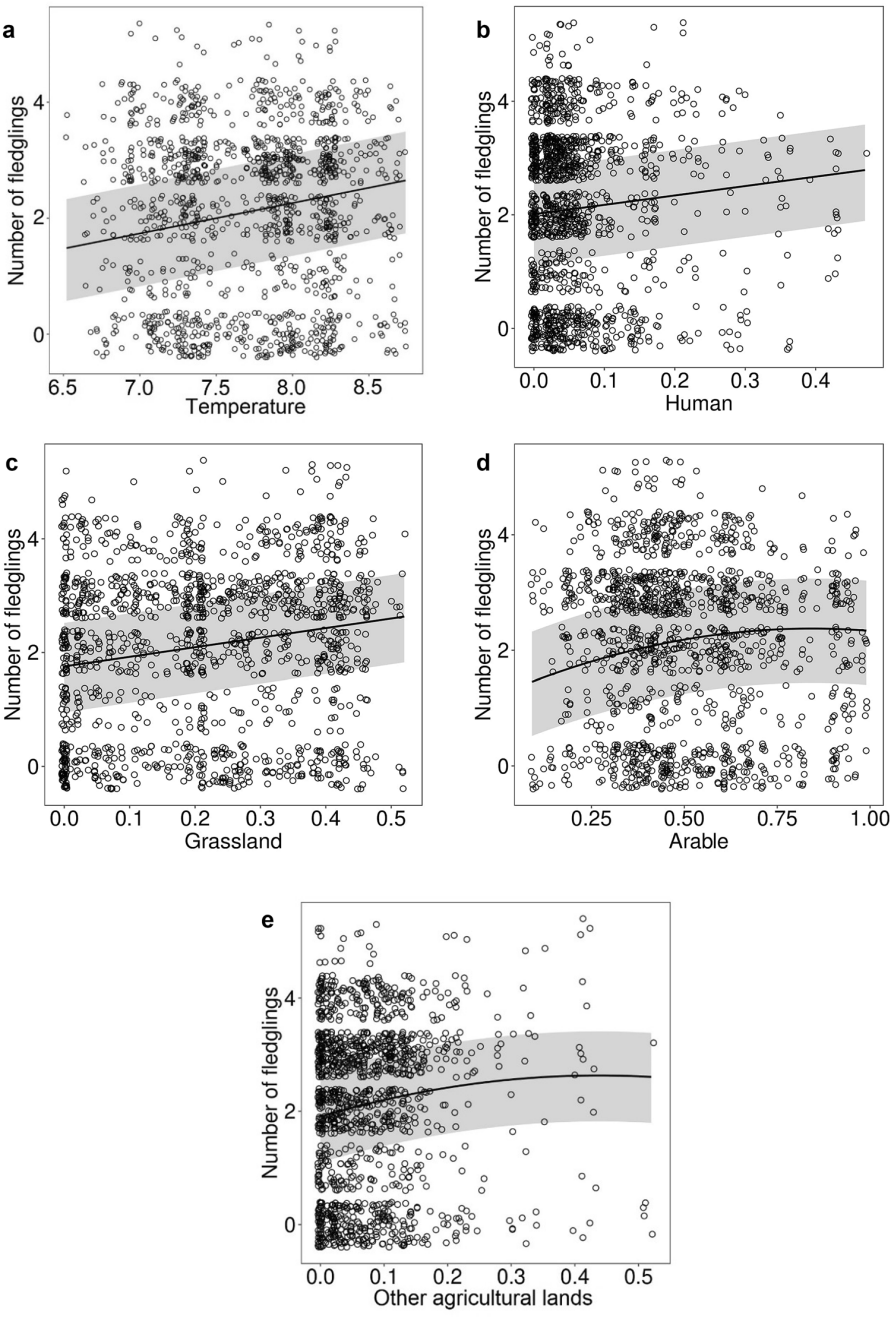
The relationship between number of fledglings and its significant predictors: **(a)** average minimum temperature during breeding season, **(b)** cover of highly human changed areas, **(c)** pastures and meadows, **(d)** non-irrigated arable lands and **(e)** other agricultural lands, based on generalized linear mixed models. The grey area represents 95% CI.

Based on GLMM analysis, the significant predictors of breeding effect (number of fledglings) of white storks were: average minimum temperature (p = 0.010); cover of areas greatly altered by humans (p = 0.039); pastures and meadows (p = 0.019); non-irrigated arable lands (p = 0.037); other agricultural lands (p = 0.027) (Table 1). However, although all predictors exerted positive effects, cover of non-irrigated arable land and other agricultural lands showed non-linear effects (Fig. 4).

**Table 1:** The linear mixed effect model with restricted maximum-likelihood describing the relationship between breeding effect of white storks with land cover, weather conditions, and distance to the nearest landfill.

	Estimate	Std. error	df	t value	Pr( >|t|)
(Intercept)	− 3.96	2.14	52.97	− 1.85	0.070
tmin	0.52	0.19	21.40	2.82	0.010*
ppt	0.00	0.01	552.44	− 0.32	0.753
def	− 0.01	0.01	123.84	− 0.99	0.327
Soil	− 0.01	0.01	91.56	− 1.31	0.193
Human	1.66	0.79	169.32	2.09	0.039*
Grassland	1.69	0.71	118.24	2.38	0.019*
Arable	2.73	1.30	180.65	2.11	0.037*
Arable^2	− 1.62	1.08	185.89	− 1.50	0.134
Agri	3.32	1.49	311.04	2.23	0.027*
Agri^2	− 3.79	3.60	509.44	− 1.05	0.293
Distance	0.16	0.09	194.40	1.78	0.078

*tmin* average minimum temperature during breeding season, *ppt* average accumulated monthly precipitation during breeding season, *def* average climatic water deficit during breeding season, *soil moisture* average soil moisture at the end of each month during breeding season, *human* cover of highly human changed areas, *grassland* cover of pastures and meadows, *arable* cover of non-irrigated arable lands, *agri* cover of other agricultural lands, *distance* ln distance to the nearest landfill.

*p < 0.05.

**Figure 5:**
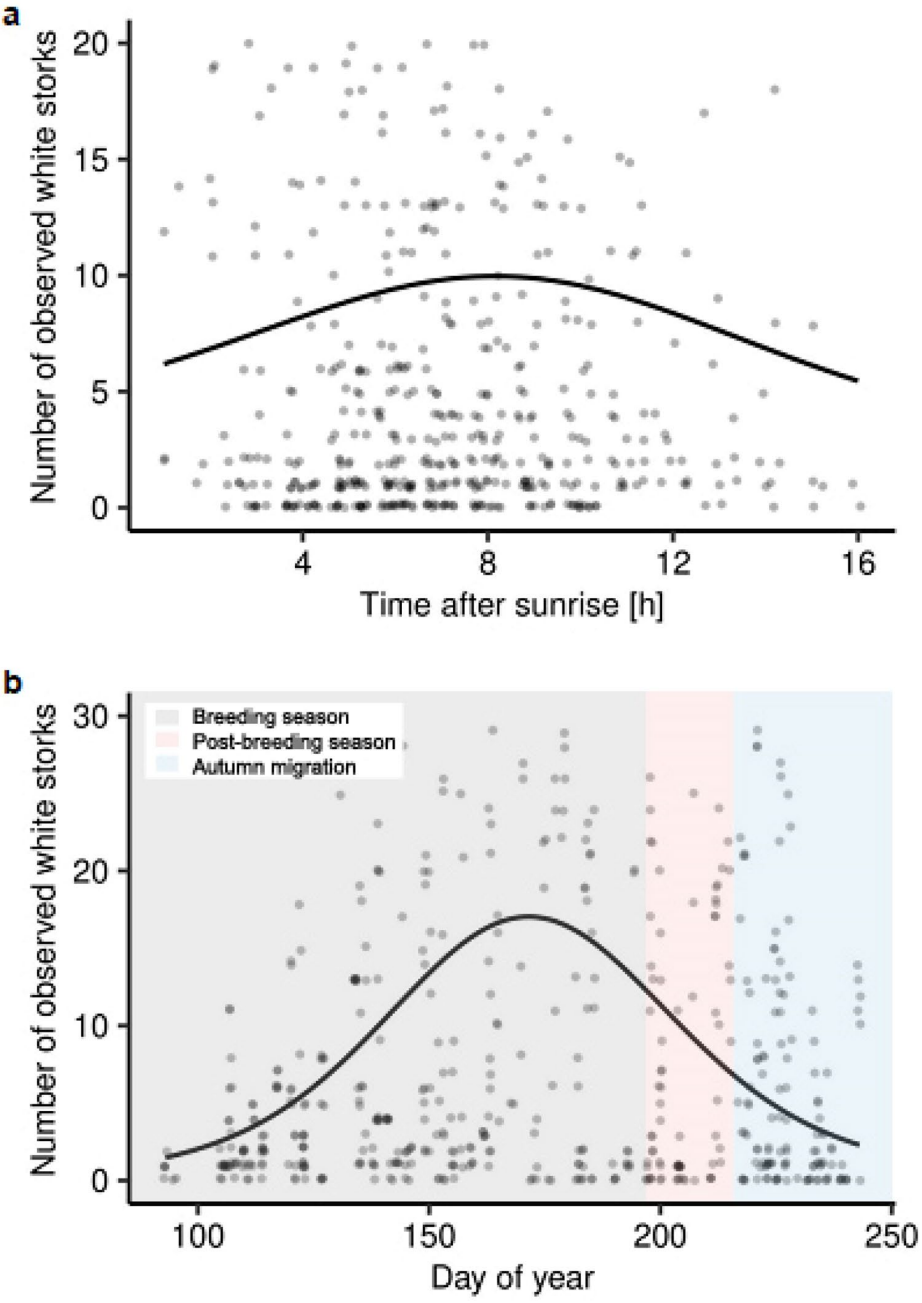
The relationship between the number of observed white storks per hour on the landfill and **(a)** time after sunrise, **(b)** day of year, based on the generalized additive mixed model.

Altogether, a maximum of 136 and an average of 9 white storks per hour were observed directly at landfills (Table 2). Based on the GAMM, we found that time after sunrise (p = 0.036) and day of year (p < 0.001) (Table 3, Fig. 5) influenced the number of white storks observed at landfills, but not the population density or landfill area. Based on the Fig. 5b white storks were the most abundant in the late breeding season. In the breeding season we observed max. 136, mean 11.2 individuals after the first year of life, in the post-breeding season max. 56, mean 8.5 individuals after the first year of life; max. 9, mean 0.3 juveniles observed per hour, and in autumn migration time max. 31, mean 4.9 individuals after the first year of life; max. 6, mean 0.1 juveniles per hour.

**Table 2:** Maximum and mean numbers of storks observed per hour on each landfill.

Landfill ID	Max		Mean	
	Juv.	Imm. + Ad.	Juv.	Imm. + Ad.
Brodnica	0	46	0.00	14.47
Czartoria	0	56	0.00	14.30
Hryniewicze	9	136	0.70	39.61
Jastrzębie-Zdrój	0	13	0.00	2.57
Łowicz-Jastrzębia	0	17	0.00	2.21
Spytkowo	6	72	0.34	17.86
Trzebania	0	2	0.00	0.62
Wawrzynki	0	36	0.00	4.50
Wola Suchożebrska	2	45	0.04	7.53

*Juv.* juvenile individuals, *Imm.* immature individuals, *Ad.* adult individuals.

**Table 3:** The GAMMs’ with the negative binomial distribution describing the relationship between number of white storks observed on the landfill per hour and population size, landfill area, time after sunrise, day of year and temperature.

Variable					
Parametric coefficients	Estimate	SE	Z-value	p	
Population density	0.03	0.02	1.80	0.072	
Landfill area	0.13	0.25	0.51	0.608	

Smooth terms	Edf	Ref. df	Chi. sq	p	
Time after sunrise	1.87	1.98	6.87	0.036	*
Day of year	1.98	2.00	119.93	< 0.001	**
Temperature	1.77	1.95	3.61	0.196	

*p < 0.05.

**p < 0.01.

## Discussion

The present study is a comprehensive research on the use of landfills as a feeding source in the breeding season of the white stork. We included long-term data from various regions, diverse in terms of land use, as opposed to many previous studies conducted on a relatively local scale^13,30,55–58^.

Our results show that land cover, which represents habitat quality, has a crucial effect on the probability of nest occupation which is consistent with previous findings^30,43^, and that the most important habitats for white storks are non-irrigated arable lands, grasslands, and other agricultural lands. Taken together, confirm that the white stork is currently a farmland bird that requires a mosaic of arable land with significant proportion of semi-natural and natural habitats. This is in contrast to the current reports from Western Europe^59^ and North Africa^60–62^ where often white storks rely greatly on anthropogenic sources of food, i.e. landfills, or even in some areas, it has become an urban species^60,63^. Moreover, distance to landfill is also of great importance. However, in one year of the study, 2016, the most important factors for nest-site selection apart from non-irrigated arable lands and pastures and meadows were weather conditions (climate water deficit, soil moisture, and precipitation), not distance to landfill. This shows that, whereas there may be a general preference for type of habitat, in particular years this may change in accordance with current weather conditions, that affect natural food availability^25,39^. Although it should be mentioned, that 10-km buffer might be insufficient to detect clear differences, while storks may sometimes perform further foraging trips^26^.

Nest-site and habitat selection in general have been shown to be affected by both land use and climate^64–69^; thus changes in farming management were particularly severe in the case of farmland bird species, many of which declined due to loss of habitats^70–73^. As opposed to land use, weather conditions are rarely considered in habitat selection studies, but are in fact drivers of seasonal variation in selecting habitat. However, they can also be addressed in terms of fine-scale temporal variation^74^.

The use of landfills, which is quite common in many species, is affecting their breeding behaviour, including nest-site selection^8^. Similarly to our results, it was shown in the case of Egyptian vultures *Neophron percnopterus* that distance to predictable anthropogenic food subsidies, including landfills, along with environmental variables, affect probability of territory occupancy^75^. As well, presence and distance to landfill were shown to be a predictor of colony growth in rooks *Corvus frugilegus*^76^.

White storks are known to use meadows exclusively in the extended part of the breeding season, whereas at the beginning and the end of this season they forage in other agricultural areas as often as in meadows^77^. This and previous studies show that, due to anthropogenic habitat modification and the degradation of semi-natural habitats such as pastures and meadows, the white stork has adapted to the use of agricultural habitats^30,78^. Proportions of pastures and meadows differ greatly between regions, and the overall percentage is low^39^. Habitat, however, is complex in terms of structure, and land cover is only one of the elements explaining breeding success. Our results clearly indicate that weather conditions and soil–water relationships may be important, as they affect prey abundance, especially in poor habitats^79^. Moreover, our results show a non-linear relationship between breeding effect and arable lands (Fig. 4d,e), which is consistent with the most recent study of Orłowski et al.^39^. Our results suggest that landscape structure is too complex to be used to predict the white stork’s breeding success.

Breeding effect, consistent with previous studies^30^, was affected by the cover of arable lands, as mentioned above, other agricultural lands, pastures and meadows, and areas greatly altered by humans, but as well by minimum temperature. It was previously shown that breeding success of the white storks is affected by weather conditions, especially low temperatures^40,79,80^. We found no significant effect of distance to landfill on breeding effect; this finding is exactly the same as that on a more regional scale^30^. There may be several reasons for the lack of effect on breeding effect. Firstly, data on breeding success (the ratio of number of fledglings to number of hatchlings or number of laid eggs) is very difficult to obtain because most nests are currently located on electric pylons^81,82^. Moreover, breeding effect, although known as the best proxy^19^, may be insufficient. Secondly, according to previous studies, abundant invertebrate prey is a critical food resource for younger nestlings^25,83^. Thus, despite occupation of nests closer to landfills, which provide alternative resources, the resulting waste food is insufficient to ensure a higher level of breeding success due to a lack of natural food resources for nestling survival at an earlier stage of development. Similar results were also obtained in Algeria, where the probability of establishing a large colony was greater in the vicinity of a landfill, but where distance to landfill did not influence breeding effect, which was affected by precipitation^9,84^. In Switzerland, in years of high reproductive output, no significant differences were noted between nests with and without supplementary feeding^85^. The lack of effect of landfill proximity on breeding effect may arise from the age of birds that use landfills. Younger animals show a high degree of behavioural plasticity and thus are less neophobic and more inclined to use new food sources^86–88^. If birds feeding at landfills are mostly younger birds, we can expect that their lack of experience will not be easily compensated by accessible food sources and thus their level of breeding success will continue to be low. It is crucial to continue direct observations on landfills along with ringing projects in order to better explain this phenomenon.

Our results show that the number of white storks observed at landfills peaks 8 h after sunrise. This suggests that white storks use other feeding areas in the early morning hours, e.g. feeding on earthworms, which are abundant in dewy meadows and fields. Then, when earthworm abundance decreases with rising temperatures and air thermals are conducive to flying long distances, storks use landfills as food sources.

We also found that the white stork uses landfills mostly in the middle of the breeding season (late June), the time when the food demands of nestlings are greatest^25^. Once chicks are fledged, numbers of white storks at landfills decrease, which is in contrast to results from Western Europe where landfills are used by storks intensively after breeding season, during autumn migration^26,89^. Surprisingly, we found no relationship between numbers of white storks and population density. This may result from the small percentage of the population that feeds on landfills. The maximum of 136 birds observed at landfills is actually small compared to western European countries, where hundreds or even thousands of storks feed at landfills^55^. Furthermore, as the peak in the number of visiting birds is observed in late June, it is certain that at this time we observe not only breeding birds but also non-breeders, which are also at the peak of their abundance^90^. If some visiting storks are non-breeders, then it confirms our hypothesis concerning the absence of an effect of landfill proximity on breeding effect. This would be consistent with the findings of Gilbert et al.^26^ that landfills are used more during non-breeding season (although resident storks may use landfills due to the scarcity of natural food sources during non-breeding season, this may be caused as well by the specific nutritional demands of chicks), as well as with examples from the eastern migration route, where landfills are used intensively^28^. This is also consistent with studies of other species, e.g. black kites *Milvus migrans,* which feed at landfills mainly during migration or as non-breeders, but rarely use landfills while breeding^91^. Although white storks have been observed at landfills in CEE for over two decades^29^, this phenomenon has not developed on a scale similar to that of the Western European population of white storks. One potential explanation is that the level of naturalness of agricultural land is still high in this part of Europe and white storks have not been forced to change their feeding habits. What is more, landfills are also a source of many pathogens for foraging birds^16,63,92^, as well as of toxins^15,17,18^ that may reduce hatchling survival and, eventually, fitness. To defend themselves from pathogens and toxins, individuals need additional energy resources, as the immunological system is costly^93^. In warmer climates, metabolisation of pathogens may be easier due to the lower cost of thermoregulation. In CEE the climate may sometime be severe for breeding birds and may reduce breeding success^40^. Therefore, in CEE, white storks which use landfills as foraging grounds may not achieve higher levels of breeding success than those foraging more naturally.

It seems that feeding at landfills is still facultative, and the trend of nesting closer to landfills^30^ does not determine breeding effect. Furthermore, numbers of individuals observed at the landfills are not related to breeding densities; the peak of storks’ abundance on landfills occurs in late breeding season, suggesting that some of the birds using landfills are non-breeders. Based on above, the planned open-air landfills closing in European Union probably will not affect population trends of CEE white stork. However, there are still many unresolved questions, concerning not only the consequences of feeding at landfills but also how this novel behaviour is learned. The importance of feeding at landfills in CEE countries should be studied further, during both breeding and non-breeding seasons. The slow progress of this phenomenon in CEE still offers great potential for further studies and is certainly worth monitoring.

## Supplementary Information

Supplementary Table S1.
