# Supplementary material for: Impact of land cover and landfills on the breeding effect and nest occupancy of the white stork in Poland
Source: Sci Rep. 2021 Mar 31;11:7279. doi: 10.1038/s41598-021-86529-z (PMC8012577; doi:10.1038/s41598-021-86529-z)
Supplement: Supplementary file 1 — Supplementary Table S1. [file 41598_2021_86529_MOESM1_ESM.docx]

**Impact of land cover and landfills on the breeding effect and nest occupancy of the white stork in Poland**

Joanna T. Bialas,^1*^ Łukasz Dylewski,^2^ Andrzej Dylik,^3^ Tomasz Janiszewski,^4^ Ireneusz Kaługa,^5^ Tomek Królak,^6^ Robert Kruszyk,^7^ Krzysztof Pawlukojć,^8^ Zuzanna Pestka,^9^ Michał Polakowski,^10^ Adam Zbyryt,^11,12^ Marcin Tobolka^1,13^

^1^Institute of Zoology, Poznań University of Life Sciences, Wojska Polskiego 71C, 60-625 Poznań, Poland

^2^Institute of Dendrology, Polish Academy of Sciences, Parkowa 5, 62-035 Kórnik, Poland

^3^Kotwicowa 15, 85-435 Bydgoszcz, Poland

^4^Department of Biodiversity Studies and Bioeducation, Faculty of Biology and Environmental Protection University of Łódź, Banacha 1/3, 90-237 Łódź, Poland

^5^Grupa EkoLogiczna, B. Chrobrego 15/83, 08-110 Siedlce, Poland

^6^Towarzystwo Przyrodnicze "ALAUDA", M. Skłodowskiej-Curie 65, 87-100 Toruń, Poland

^7^Piotrowicka 52A, 44-341 Skrbeńsko, Poland

^8^Królowej Jadwigi 18C/5, 11-500 Giżycko, Poland

^9^Vertebrate Ecology and Zoology Unit, University of Gdańsk, Bażyńskiego 8, 80-309 Gdańsk, Poland

^10^Institute of Biology, University of Szczecin, Wąska 13, 71-412, Szczecin, Poland

^11^The Polish Society for Bird Protection (PTOP), Ciepła 17, 15–471 Białystok, Poland

^12^Faculty of Biology, University of Białystok, Ciołkowskiego 1J, 15-245 Białystok, Poland

^13^Konrad Lorenz Institute of Ethology, Veterinary Medicine University of Vienna, Savoyenstraße 1, 1160 Wien, Austria

*The White Stork Research Group*

*Corresponding author. E-mail address: jtwozna@gmail.com (J.T.Bialas)

Supplementary Information

Table S1 The result of the model selection.

| Model | df | lohLik | AICc | delta | weight |
| --- | --- | --- | --- | --- | --- |
| **Intercept + agri + agri^2^ + arable + arable^2^ + def + grassland + human + Distance + ppt + sol +** tmin | **16** | **-2300.973** | **4634.4** | **0.00** | **0.634** |
| Intercept + agri + agri^2^ + arable + def + grassland + human + Distance + ppt + soil + tmin | 15 | -2303.080 | 4636.5 | 2.17 | 0.215 |
| Intercept + agri + arable + arable^2^ + def + grassland + human + Distance + ppt + soil + tmin | 15 | -2303.719 | 4637.8 | 3.44 | 0.113 |
| Intercept + agri + arable + def + grassland + human + Distance + ppt + soil + tmin | 14 | -2305.823 | 4640.0 | 5.60 | 0.038 |
|  |  |  |  |  |  |
